# Simple 3D Printed Scaffold‐Removal Method for the Fabrication of Intricate Microfluidic Devices

**DOI:** 10.1002/advs.201500125

**Published:** 2015-07-16

**Authors:** Vittorio Saggiomo, Aldrik H. Velders

**Affiliations:** ^1^ Laboratory of BioNanoTechnologyWageningen UniversityPO Box 80386700EKWageningenThe Netherlands; ^2^ Laboratory of BioNanoTechnologyWageningen UniversityPO Box 80386700EKWageningenThe Netherlands; ^3^ Instituto Regional de Investigacion Cientifica Aplicada (IRICA)Universidad de Castilla‐La Mancha13071Ciudad RealSpain

**Keywords:** 3D printing, microfluidics, microfabrication, devices, NMR

## Abstract

**An easy and cheap fabrication method for intricate polydimethylsiloxane microfluidic devices** is presented. The acrylonitrile butadiene styrene scaffold‐removal method uses cheap, off‐the‐shelf materials and equipment for the fabrication of intricate microfluidic devices. The versatility of the method is proven by the fabrication of 3D multilayer, ship‐in‐a‐bottle, selective heating, sensing, and NMR microfluidic devices. The methodology is coined ESCARGOT: Embedded SCAffold RemovinG Open Technology.

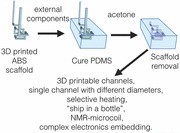

Microfluidics^1^, ^2^ is a continuously growing field, of great interest in chemistry,^3^ physics,^4^, ^5^ drug discovery,^6^ biology,^7^, ^8^ chemical biology,^9^ biomedical research,^10^ tissue engineering,^11^ and most recently, organs‐on‐chip.^12^ The small amounts of liquid required for experiments, the physics of fluids at the micro domain and the lab‐on‐chip approach make microfluidics one of the interdisciplinary field par excellence.^13^ To date, polydimethylsiloxane (PDMS) is the most popular material in research laboratories for the fabrication of microfluidic devices.^14^, ^15^, ^16^ It is relatively cheap and easy to manipulate, gas permeable and has a refractive index of 1.4, close to the one of glass. For the manufacturing of microfluidic PDMS devices, generally a master is needed, usually obtained by clean‐room lithography of silicon wafers. Consecutively, PDMS is poured on the master, and after curing, the rubber must be carefully peeled off from the master and subsequently chemically bonded to another surface after activation with oxygen plasma or using chemical solutions.

Notwithstanding the great potential, two main bottlenecks inhibit a more widespread use of PDMS devices. First, the PDMS fabrication method is considered too complex for many scientists without any experience in microfabrication.^9^ Second, achieving a 3D (multilevel channels or a single channel with different sizes) using standard fabrication methods is rather complicated, as multiple layers of PDMS must be fabricated and then sealed together to create an internal 3D channel.^17^ In recent years, sacrificial mold or fugitive ink is used for fabricating PDMS microfluidic devices. Although the use of sacrificial mold is a step forward in simplifying the fabrication of microfluidic devices, it still requires either harsh condition like the use of high temperatures for creating,^18^ or removing,^19^ a template, applying heavy swelling for pulling out the template,^20^, ^21^ or the use of complex mold fabrication such as using chitosan 22 or isomalt printed with an heavily modified 3D printer and backfilled with epoxy resin.^23^

Recently, 3D printing has been used either to print masters for soft lithography mold,^24^ or to directly print microfluidic devices.^25^, ^26^, ^27^ In the first case, although the mold is easily printed, it has the same limitation for creating multilayer and complex microfluidic devices than the standard clean room lithography. The limitation of 3D printing directly the microfluidic devices lies mainly in the material used and, so far only one example of 3D printed PDMS membrane is present in literature with the limitation of using PDMS mixed with colored photoresist, thus not pure PDMS and giving non transparent devices.^28^ PDMS is usually preferred over other 3D printing plastics because of a) its gas permeability, useful in biology for keeping cells and bacteria alive for long time in the microfluidic chip; b) its elasticity, capable of making micro pumps and valves in the device and c) its simple chemical modification using well known silane chemistry, difficult thing to do on 3D printing plastics and d) its transparency. Moreover, embedding other functionalities as described in this research, is extremely hard or even impossible using a 3D printer for directly printing a microfluidic chip.

Here we present an easy two‐step acrylonitrile butadiene styrene (ABS) scaffold‐removal method for achieving 3D, multilayer, intricate, micrometric channels in a single block of PDMS. We also show how, using the scaffold‐removal fabrication method, external components, such as heating elements, electronics or RF circuitry, can be embedded directly in microfluidic devices. A most striking example is the fabrication of a high‐resolution nuclear magnetic resonance device that provides molecular analysis of just microliter volumes. Using the ABS scaffold‐removal method, there is no need of lithography steps nor silicon masters, no need of bonding the PDMS on surfaces nor of repetitive procedures for obtaining multilevel channels, making the fabrication of microfluidic devices easy, low‐cost and opening up the field for a plethora of scientists working in different areas. We baptize this methodology ESCARGOT: Embedded SCAffold RemovinG Open Technology.

In order to avoid the use of silicon masters and (clean room) lithography, and the subsequent bonding of PDMS to another surface or the complex fabrication of sacrificial molds, we propose the use of an off‐the‐shelf plastic polymer, used for 3D printing, as scaffold for creating micrometric sized channels. The scaffold plastic polymer can be inserted into liquid PDMS, and after curing of the latter, dissolved using a PDMS‐inert solvent, leaving an empty cavity inside the PDMS (**Figure** **1**, and Video S1, Supporting Information). With this method, any 3D channel structure, even extremely intricate ones, can be created in two easy steps, basically without knowledge of, or experience in, microfabrication or lithography. In addition to the simple fabrication method we also show how, using this method, it is easily possible to integrate external components such as UV‐LED, heating unit, ship‐in‐a‐bottle, and even a fully functional NMR microcoil.

**Figure 1:**
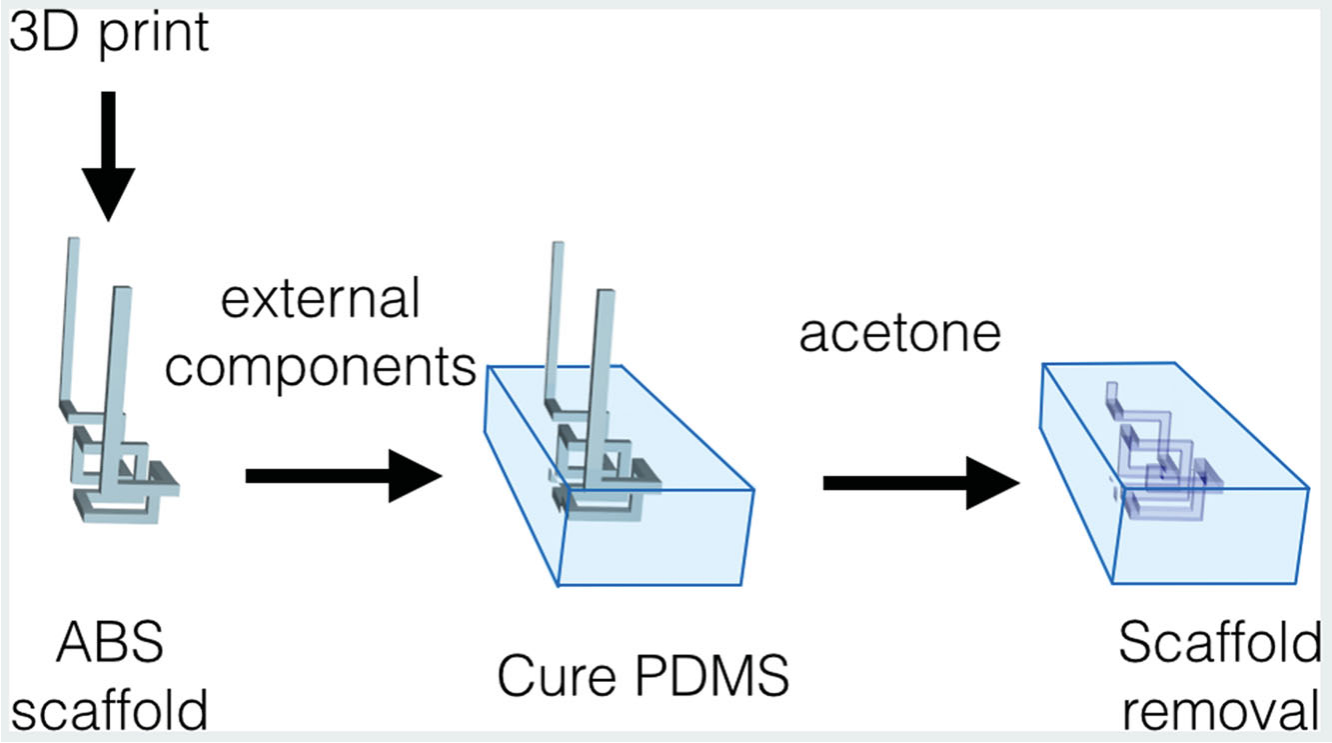
Schematic representation of the ABS scaffold‐removal fabrication method for manufacturing microfluidic devices. An ABS plastic scaffold is modeled, or 3D printed, in the desired shape (left). Consecutively, it is suspended in PDMS with or without the addition of external components and then the polymer is cured (middle). Finally, the scaffold is removed by immersion in acetone creating the microfluidic channels (right).

One of the most common plastics used for the fused deposition modeling (FDM) 3D printing, is the cheap (less than 20¤ per kg) and commercially available ABS.^29^ We extruded ABS plastic with the aid of a 500 μm nozzle giving filaments of approximately the same diameter. These scaffolds were then suspended into liquid PDMS and the latter was cured at 75 °C for 2 h, after which it was immersed in acetone for 12 h, dissolving the scaffolds. Acetone was the solvent of choice for dissolving ABS whilst its swelling ratio (S) for PDMS is as low as 1.06.^30^ A final flushing with acetone completely cleaned the inner channel, creating de facto a PDMS microfluidic device. Changing the nozzle of an off‐the‐shelf 3D printer to nozzle with smaller diameter of 400, 300, and 200 μm is easy and provides microfluidic channels of the same diameter (Figures S2 and S3, Supporting Information). It is not hard to imagine that, giving the rise of commercially available 3D printers, in the next year nozzles with diameters of about 100 μm or even smaller will hit the market. At those scales, roughness of few micrometers is non influential for the performance of the microfluidic chip, and this roughness is comparable to commercially available sandblasted glass microfluidic chips. Although the roughness due to the nozzle is in the order of few micrometers, the one coming from the layer by layer ABS deposition for more complex designs is much higher and it is dependent by the resolution of the printer, spanning from 100 to 10 μm.

Many different 3D channels were readily created using the ABS scaffold‐removal method (**Figure** **2**, and Video S2, Supporting Information): spiral channels (Figure 2a), multichannels with different geometries (Figure 2b) and channels with compartments differing in size (Figure 2c). As further proof of concept, a complex 3D multilevel scaffold based on the Hilbert curve^31^ was designed and 3D‐printed utilizing ABS fuse deposition modeling. Also in this extreme case, with the single channel inside the PDMS having a length of 35 cm and containing 1.4 cm^3^ of ABS, it was still possible to remove the plastic with subsequent baths in dichloromethane and acetone (Figure 2d).

**Figure 2:**
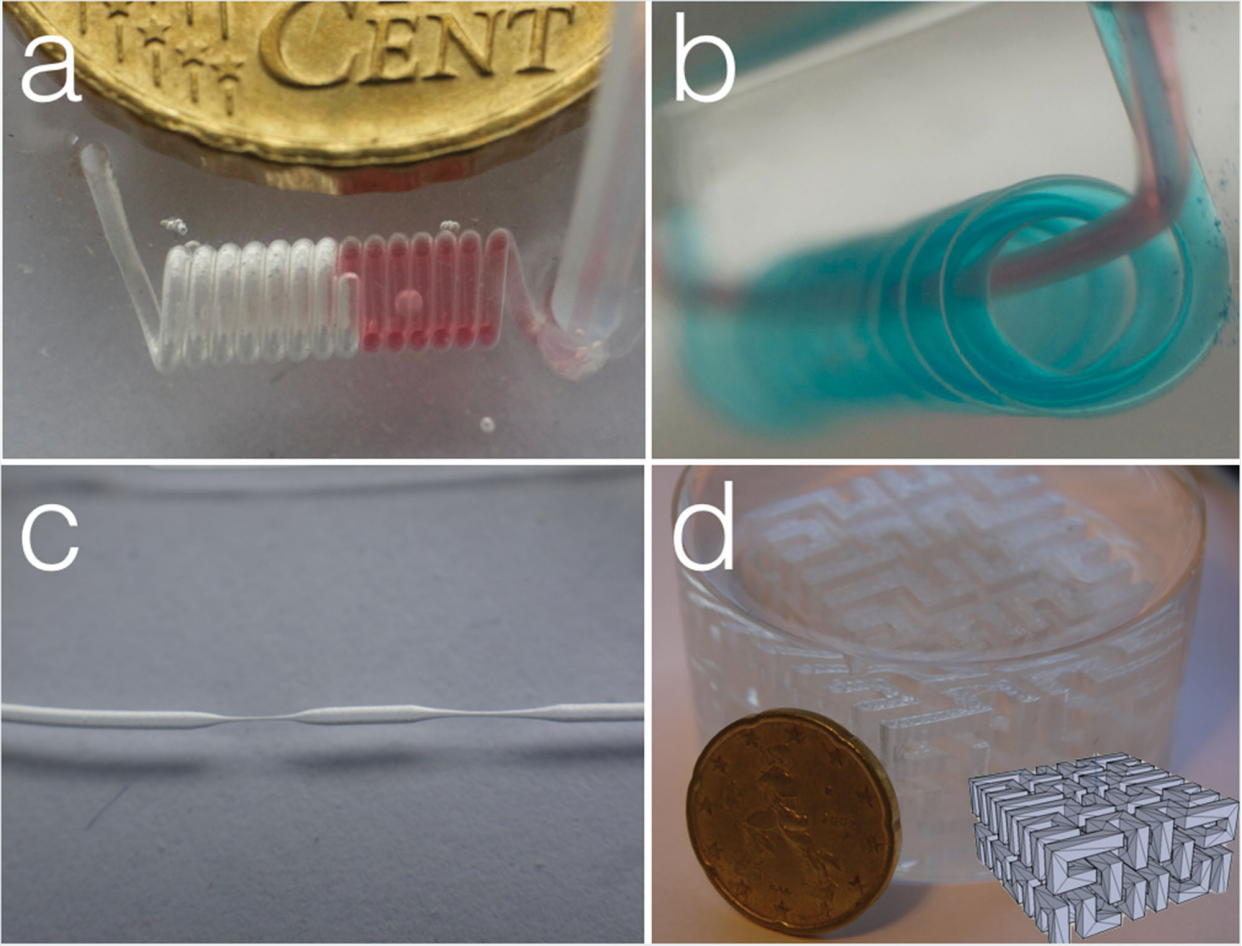
Various 3D multilayer PDMS microfluidic devices fabricated using the ABS scaffold‐removal method. a) Spiral microfluidic device; b) a microfluidic channel wrapped around another one; c) a single channel with different diameter and d) a microfluidic device fabricated using a 3D printed object used as scaffold. Diameter of the channels is 500 μm in (a) and (b), 500 μm and 90 μm in (c), and 2 mm in (d).

Integrating external elements directly in the microfluidic device is desirable for lab‐on‐a‐chip approaches but difficult to achieve using standard PDMS fabrication methods. We incorporated stirring bars, electronic circuitry, heating elements, and radiofrequency (RF) components, illustrating the wide and versatile applicability of this method (**Figure** **3**, and Video S3, Supporting Information).

**Figure 3:**
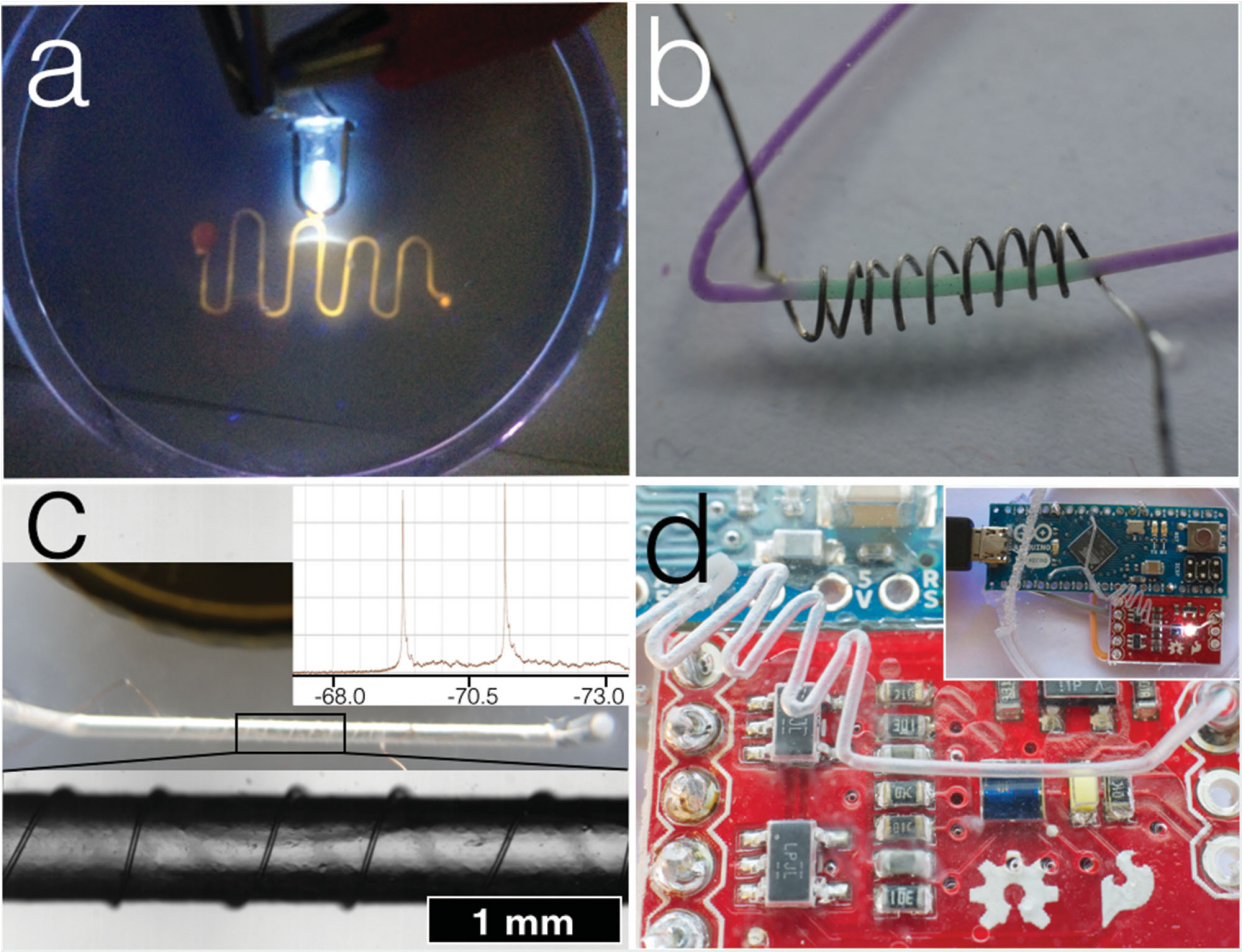
Various electronic components embedded in microfluidic chips. a) a 390 nm UV‐LED illuminating a fluorescent dye in the channel; b) a resistance wire, used as selective heating unit; a thermochromic dye changes color only where the resistance wire is coiled around the channel; c) a 32 μm copper wire wrapped around a microfluidic channel is used as solenoidal microcoil allowing high‐resolution NMR spectroscopy on 2 μL sample volumes; the insert shows the ^31^P‐coupled ^19^F spectrum of a NaPF_6_ solution in water, values in ppm; d) a fully functional Arduino microcontroller coupled with a color sensor embedded into the PDMS chip. The diameter of the channels in all the pictures is 500 μm.

External components in a cavity bigger than the size of the channels guiding to it, so‐called “ship‐in‐a‐bottle,”^32^ can be included in the ABS polymer and subsequently released during the acetone treatment. In this way a small cylindrical 1 × 1 mm magnet was inserted in a microfluidic chamber (Video S3 and Figure S11, Supporting Information).

As PDMS is nonconductive (resistivity 10^13^–10^15^ Ω/cm) and acetone is a noncorrosive solvent, electronic components can also be embedded directly in the design. These can be simply inserted in the PDMS together with the microchannel scaffolds before curing it, then the acetone treatment removes only the scaffold leaving the electronics intact. In this way we inserted a 390 nm LED for the optical detection or electronic excitation of chemicals in the microfluidic channel (Figure 3a).

Another problem usually associated with microfluidic chip is the difficulty of heating only part of the channel inside the chip. Taking advantage of PDMS its low thermal conductivity (0.15 W m^−1^ K) we envisioned a selective heating unit inside a microfluidic device. A 200 μm Nichrome resistance wire was loosely wrapped around the ABS polymer scaffold and inserted in PDMS. After the curing step and dissolving the ABS scaffold, a voltage of 1.2 V with 0.35 A sufficed for selectively heating a thermochromic dye above 27 °C only in the part of the channel surrounded by the resistance wire (Figure 3b, and Video S3, Supporting Information). Temperatures can be varied and the 200 μm wire allows, for example, to boil water inside the channel (Video S3, Supporting Information). This simple and selective heating element embedded in the microfluidic chip can be of great value for designing chips to perform, e.g., biological experiments such as PCR, sterilization inside the microchannels or for setting different temperatures for organ‐on‐chips or cell cultures.

NMR spectroscopy is arguably one of the most powerful analytical tools available to the scientific community. However, NMR is a notoriously insensitive technique because of the unfavorable Boltzman distribution of the spin states, severely compromising analysis of mass‐ and volume‐limited samples, like in microfluidics. Approaches to solve sensitivity issues comprise most expensive and technologically demanding solutions as using extreme magnetic field strengths and complex NMR probe techniques.^33^ Alternatively, down‐scaling the RF transceiver coils to match the size of the sample significantly increases the sensitivity,^34^ although these small‐volume probes still require technological demanding fabrication methods.^35^ We decided to exploit the concept of ABS scaffold‐removal to make a simple and cheap, yet most sensitive NMR sensor. A 32 μm copper wire was wrapped around a 500 μm ABS filament, resulting in a final channel encompassed by a solenoidal NMR microcoil (Figure 3c), with a detection volume of only two μL; normal NMR tubes contain about 500 μL sample volume. This microfluidic device was integrated on a cylindrical aluminum probe insert and placed inside a 9.4 T narrow‐bore superconducting NMR magnet. Tuning the resonance circuit to 376 MHz, straightforwardly allowed high‐resolution NMR spectra to be obtained (Figure 3c insert). Line‐widths at half peak‐height were obtained of about 3 Hz and resolving heteronuclear spin–spin couplings, opening up the way to further optimization and applications (Figures S15 and S16, Supporting Information). The lines of the doublet observed in the ^31^P‐coupled ^19^F‐spectrum of a 1 m NaPF_6_ sample correspond to an amount of one micromole of spins that is detected. This amount is in the order of the lower mM concentrations measurable in conventional NMR probes; further optimization of the probe coil is currently ongoing to allow for an increased concentration as well as mass sensitivity. In addition, we calculated that the material costs for fabricating this device is less than two euro. Thus, the microfluidic NMR‐device, in which sample container and transceiver coil are integrated, is cheaper than a standard NMR tube, and orders of magnitude cheaper than an NMR probe head worth several thousand euro.

Last proof of the versatility of the ABS scaffold‐removal method was the embedding of a fully functional color sensor and a microcontroller directly in a microfluidic device (Figure 3d). An Arduino micro and a color sensor were wired together and immersed in PDMS with an ABS scaffold. After curing the PDMS and removing the ABS polymer with acetone, the resulting microfluidic channel was right on top of the color sensor. Hooking up the Arduino to a computer revealed all the components of the microcontroller and the sensor to be working properly.

Thus, even complex electronics can be easily embedded in PDMS microfluidic devices reducing the amounts of external components needed for using, screening and analyzing experiments in microfluidics devices.

Although in its infancy, where roughness and channel sizes can still be improved by the ongoing 3D printing technology, the ABS scaffold‐removal method is simple and cheap compared to the current fabrication methods, yet powerful and versatile in creating 3D multilevel and intricate microfluidic channels in PDMS. Moreover additional elements like heating coils, RF circuitry or electronic components can be embedded directly, opening up new windows for the various fields of microfluidics and PDMS devices. Creating multilevel 3D microfluidic devices can be of benefit in many different fields, for example in fabricating complex vascular systems for organ on chips, or in handling spherical droplets in tubular channels of different sizes or in mixing liquids. Because of the multidisciplinary fields of applications we can envision, the simplicity of this method, coined ESCARGOT, is of great value for all scientists willing to work with microfluidic devices, regardless of their background.

## Experimental Section

SYLGARD silicone elastomer 184 and SYLGARD silicone elastomer 184 curing agent were obtained by Dow Corning Corporation. A 3D SIMO pen was used for extruding 1.7 mm ABS, plastic filament that was obtained from the same vendor. 3D print of Hilbert cube was ordered online and 3D printed by ridix.nl (Rotterdam, the Netherlands) using a Dimension SST 1200es printer and by 3dhubs.com using a Duplicator 4 printer.

Acetone was obtained from Sigma‐Aldrich. Sparkfun RGB color sensor ADJD311 was bought from sparkfun.com, thermochromic dye from mindsetsonline.co.uk, Arduino micro and 200 μm Nichrome resistance wire from rs‐components.

The ABS plastic filament was extruded through a 500 μm nozzle (3D SIMO pen) or a Craftbot 3D printer with 400, 300, and 200 μm nozzle and then modeled into the desired 3D shape with the help of a soldering iron set (100 °C) or printed with a fused deposition modeling 3D printer. The modeled ABS plastic was then immersed in a well mixed solution of 10:1 sylgard 184/sylgard 184 curing agent. The PDMS was then placed under vacuum for removing air bubbles and cured for 2 h at 75 °C, or overnight at room temperature. The PDMS was consecutively left for 12 h in acetone, after which the microchannels were cleaned with acetone and dried with a flow of compressed air.

NMR spectroscopy was performed on an Oxford Instruments 9.4 T superconducting magnet, equipped with a 14‐coils shimming set‐up, interfaced with a Varian Inova spectrometer. The ABS‐NMR device was a cylindrical shaped PDMS disk of about 4 cm in diameter and 1 cm height, positioned on top of an aluminum cylinder of a sacrificed NMR probe. The detection volume encompassed by the solenoid sums up to ≈2 μL. The two 32 μm ends of the copper solenoid wire were soldered to two connection leads with a variable (3–18 pF) capacitor in parallel, and wired to the tuning and matching circuit of the probe, respectively grounded, allowing the fine‐tuning of the resonance circuit to be done with the probe positioned inside the magnet. Experiments were run in non‐locked mode, typically using single‐scan acquisitions using the Vnmrj 2.2D software. For the ^19^F‐NMR experiments the coil was tuned to 376 MHz, and the acquired data were processed using Mnova (MestreLab, Santiago de Compostela, Spain).

## Supporting information

As a service to our authors and readers, this journal provides supporting information supplied by the authors. Such materials are peer reviewed and may be re‐organized for online delivery, but are not copy‐edited or typeset. Technical support issues arising from supporting information (other than missing files) should be addressed to the authors.

Supplementary

Supplementary

Supplementary

Supplementary

## References

[1] P. Nge , C. Rogers , A. Woolley , Chem. Rev. 2013, 113, 2550. 23410114 10.1021/cr300337x PMC3624029

[2] G. Whitesides , Nature 2006, 442, 368. 16871203 10.1038/nature05058

[3] K. Elvira , X. Solvas , R. Wootton , A. de Mello , Nat. Chem. 2013, 5, 905. 24153367 10.1038/nchem.1753

[4] H. Stone , S. Thutupalli , Nat. Phys. 2014, 10, 87.

[5] T. Squires , S. Quake , Rev. Mod. Phys. 2005, 77, 977.

[6] P. Neuži , S. Giselbrecht , K. Länge , T. Huang , A. Manz , Nat. Rev. Drug Discovery 2012, 11, 620. 22850786 10.1038/nrd3799 PMC6493334

[7] D. Beebe , G. Mensing , G. Walker , Annu. Rev. Biomed. Eng. 2002, 4, 261. 12117759 10.1146/annurev.bioeng.4.112601.125916

[8] S. K. Sia , G. Whitesides , Electrophoresis 2003, 24, 3563. 14613181 10.1002/elps.200305584

[9] Q. Zhang , R. Austin , J. Bionanosci. 2012, 4, 277. 10.1007/s12668-012-0051-8 PMC3546818 23336098

[10] E. Sackmann , A. Fulton , D. Beebe , Nature 2014, 507, 181. 24622198 10.1038/nature13118

[11] N. Choi , M. Cabodi , B. Held , J. P. Gleghorn , L. J. Bonassar , A. D. Stroock , Nat. Mater. 2007, 6, 908. 17906630 10.1038/nmat2022

[12] S. Bhatia , D. Ingber , Nat. Biotechnol. 2014, 32, 760. 25093883 10.1038/nbt.2989

[13] D. Mark , S. Haeberle , G. Roth , F. Stetten , R. Zengerle , Chem. Soc. Rev. 2010, 39, 1153. 20179830 10.1039/b820557b

[14] J. McDonald , G. Whitesides , Acc. Chem. Res. 2002, 35, 491. 12118988 10.1021/ar010110q

[15] K. Ren , Y. Chen , H. Wu , Current Opin. Biotechnol. 2014, 25, 78. 10.1016/j.copbio.2013.09.004 24484884

[16] K. Ren , J. Zhou , H. Wu , Acc. Chem. Res. 2013, 46, 2396. 24245999 10.1021/ar300314s

[17] H. Wu , T. Odom , D. Chiu , G. Whitesides , J. Am. Chem. Soc. 2003, 125, 554. 12517171 10.1021/ja021045y

[18] J. Lee , J. Paek , J. Kim , Lab Chip 2012, 12, 2638. 22699280 10.1039/c2lc40267j

[19] S.‐H. Song , C.‐K. Lee , T.‐J. Kim , I. Shin , S.‐C. Jun , H.‐I. Jung , Microfluid. Nanofluid. 2009, 9, 533.

[20] M. Verma , A. Majumder , A. Ghatak , Langmuir 2006, 22, 10291. 17107035 10.1021/la062516n

[21] Y. Jia , J‐H. Jiang , X.‐D. Ma , Y. Li , H.‐M. Huang , K.‐B. Cai , S.‐X. Cai , Y.‐P. Wu , Chin. Sci. Bull. 2008, 53, 3928.

[22] J. G. Fernandez , J. Samitier , C. A. Mills , J. Biomed. Mater. Res. A 2011, 98A, 229. 10.1002/jbm.a.33038 21548017

[23] M. K. Gelber , R. Bhargava , Lab Chip 2015, 15, 1736. 25671493 10.1039/c4lc01392a PMC4480337

[24] K. Kamei , Y. Mashimo , Y. Koyama , C. Fockenberg , M. Nakashima , M. Nakajima , J. Li , Y. Chen , Biomed. Microdev. 2015, 17, 36. 10.1007/s10544-015-9928-y 25686903

[25] P. J. Kitson , M. H. Rosnes , V. Sans , V. Dragone , L. Cronin , Lab Chip 2012, 12, 3267. 22875258 10.1039/c2lc40761b

[26] J. L. Erkal , A. Selimovic , B. C. Gross , S. Y. Lockwood , E. L. Walton , S. McNamara , R. S. Martin , D. M. Spence , Lab Chip 2014, 14, 2023. 24763966 10.1039/c4lc00171k PMC4436701

[27] A. I. Shallan , P. Smejkal , M. Corban , R. M. Guijt , M. C. Breadmore , Anal. Chem. 2014, 86, 3124. 24512498 10.1021/ac4041857

[28] T. Femmer , A. J. C. Kuehne , M Wessling , Lab Chip 2014, 14, 2610. 24828586 10.1039/c4lc00320a

[29] H. Lipson , M. Kurman , Fabricated: The New World of 3D Printing, Wiley, Indianapolis, IN, USA 2013.

[30] J. Lee , C. Park , G. Whitesides , Anal. Chem. 2003, 75, 6544. 14640726 10.1021/ac0346712

[31] D. Hilbert , Math. Ann. 1870, 1.

[32] K. Sugioka , J. Xu , D. Wu , Y. Hanada , Z. Wang , Y. Cheng , K. Midorikawa , Lab Chip 2014, 18, 3447. 10.1039/c4lc00548a 25012238

[33] A. Bhattacharya , Nature 2010, 463, 605. 20130626 10.1038/463605a

[34] R. M. Fratila , A. H. Velders , Annu. Rev. Anal. Chem. 2011, 4, 227. 10.1146/annurev-anchem-061010-114024 21391818

[35] R. M. Fratila , M. Gomez , S. Sýkora , A. H. Velders , Nat. Commun. 2014, 5, 3025. 24394755 10.1038/ncomms4025

