# Supplementary material for: Simple 3D Printed Scaffold‐Removal Method for the Fabrication of Intricate Microfluidic Devices
Source: Adv Sci (Weinh). 2015 Jul 16;2(9):1500125. doi: 10.1002/advs.201500125 (PMC5034835; doi:10.1002/advs.201500125)
Supplement: Supplementary file 1 — Supplementary [file ADVS-2-0o-s003.pdf]

Copyright WILEY-VCH Verlag GmbH & Co. KGaA, 69469 Weinheim, Germany, 2015.

Supporting Information

for *Adv. Sci.*, DOI: 10.1002/advs.201500125

Simple 3D Printed Scaffold-Removal Method for the Fabrication of Intricate Microfluidic Devices

Vittorio Saggiomo and Aldrik H. Velders**

Supporting Information

Simple 3D printed scaffold-removal method for the fabrication of intricate microfluidic devices

Vittorio Saggiomo, Aldrik H. Velders**

Videos:

Video S1: ABS scaffold-removal method for the fabrication of microfluidic devices

Video S2: Various 3D multilayer PDMS microlfuidic devices fabricated with the ABS scaffold-removal method

Video S3: Various external components embedded in PDMS microfluidic devices using the ABS scaffold-removal method

ABS scaffold-removal for fabricating complex 3D multilayer PDMS microfluidic devices:

Figure S1,S2,S3: ABS scaffold, and different diameters channels

Figures S4,S5,S6: Various 3D multilayer PDMS microfluidic devices

Figures S7,S8: Multiple microchannels

Figure S9: Single microchannel with different diameters

Figure S10: 3D printed microchannel based of Hilber curve

ABS scaffold-removal for embedding external components in PDMS microfluidic devices:

Figure S11: "ship-in-a-bottle"

Figure S12: UV-LED embedded in a PDMS device

Figure S13: Selective heating unit embedded in a PDMS device

Figure S14,S15,S16: PDMS NMR microcoil

Figure S17,S18: Arduino and a color sensor embedded in a PDMS device

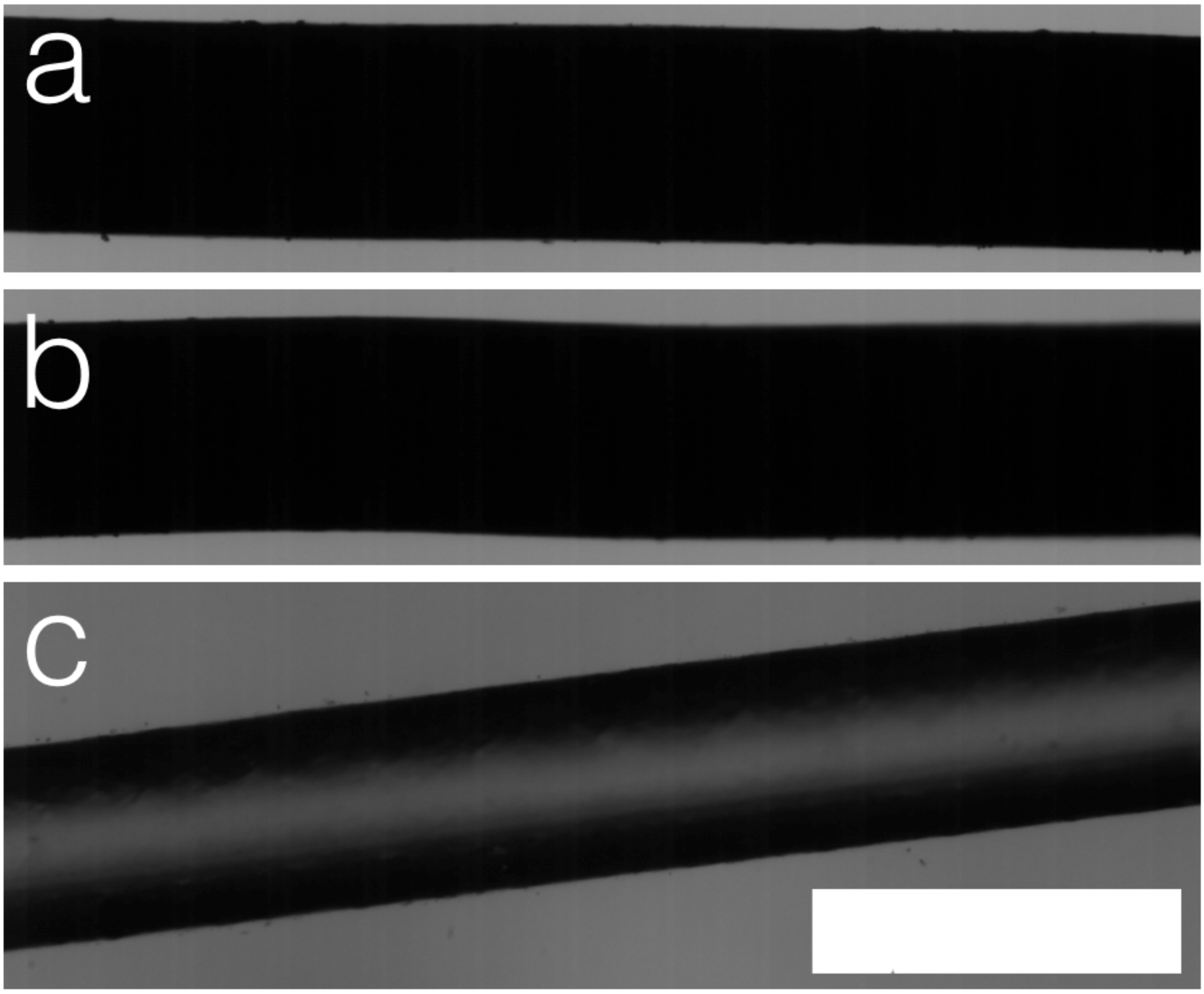

Figure S1. a) ABS plastic filament extruded from a 500 μm nozzle and b) the same ABS plastic filament inside the cured PDMS. c) Microchannel after the removal of the ABS plastic filament. Scale bar = 1 mm.

The plastic filament extruded through the nozzle of the 3D SIMO is roughly 530-560 μm measured with a bright-field microscope. The removal of the plastic filament from the cured PDMS with acetone does not affect the size nor the shape of the formed channel.

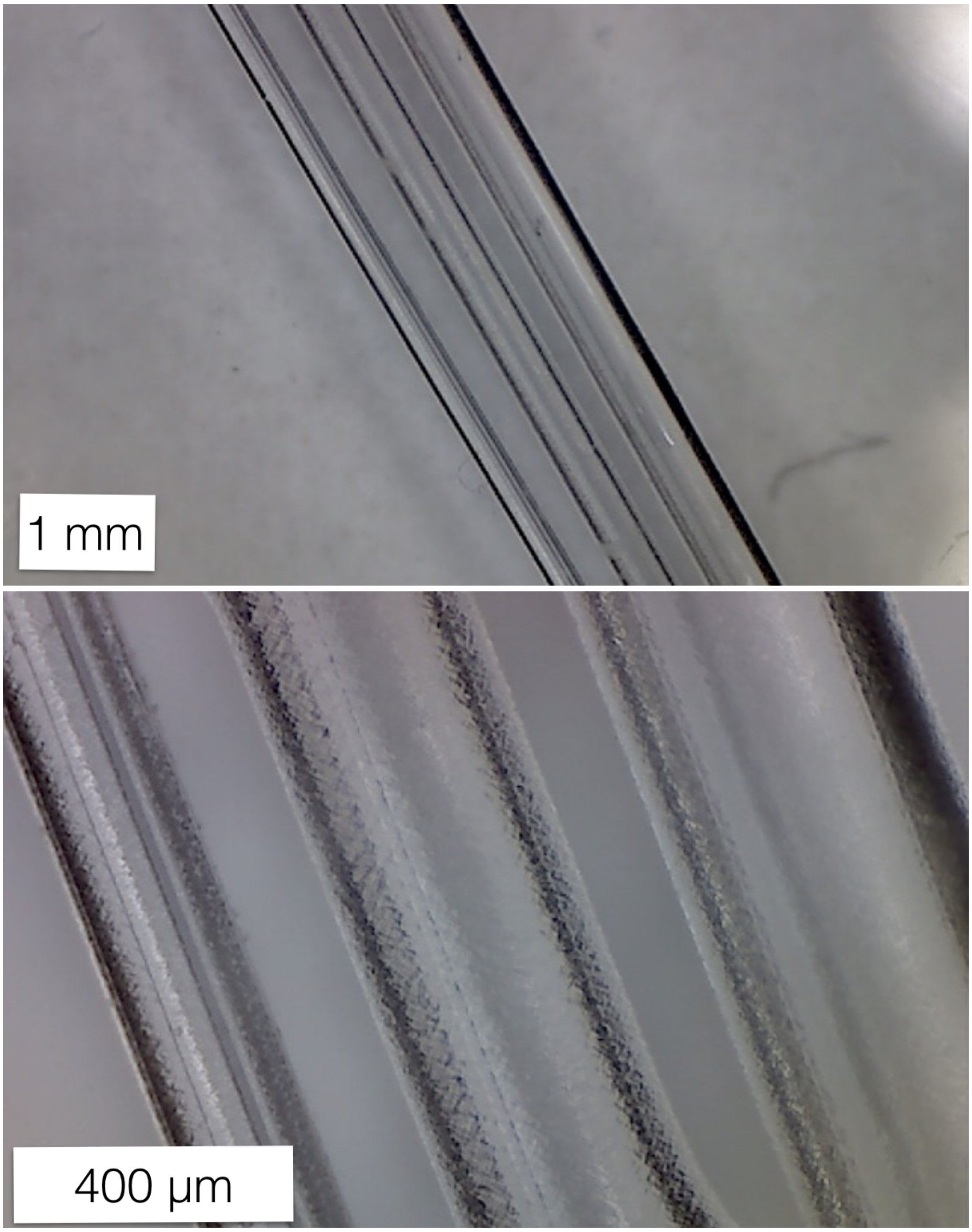

Figure S2. Top, from left to right 200 μm, 300 μm and 400 μm channels fabricated with the ABS scaffold-removal method. Bottom, same channels with higher magnification. ABS polymer was extruded via Craftbot 3D printer using nozzles of 200 μm, 300 μm and 400 μm diameters.

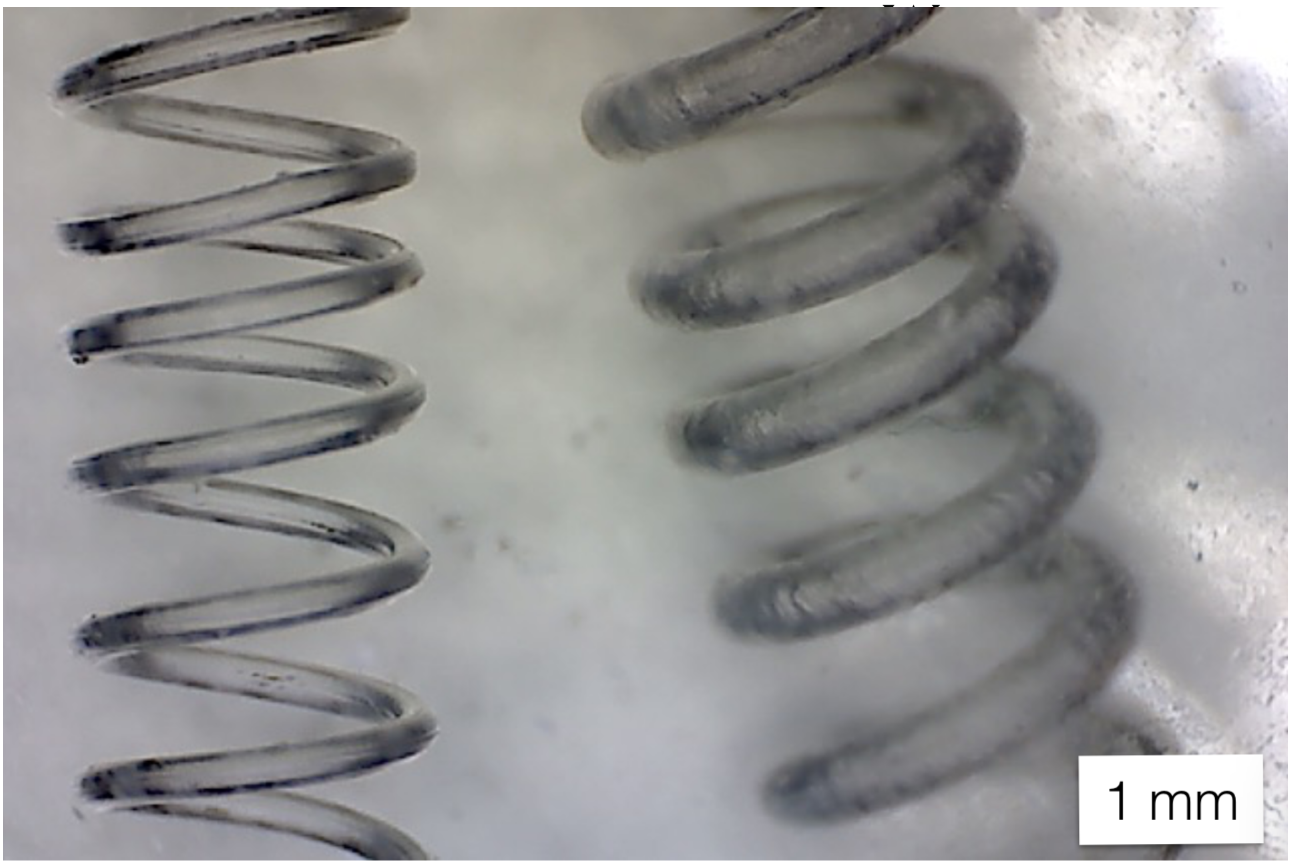

Figure S3. Two side to side spiral channel with diameter of 200 μm (left) and 400 μm (right).

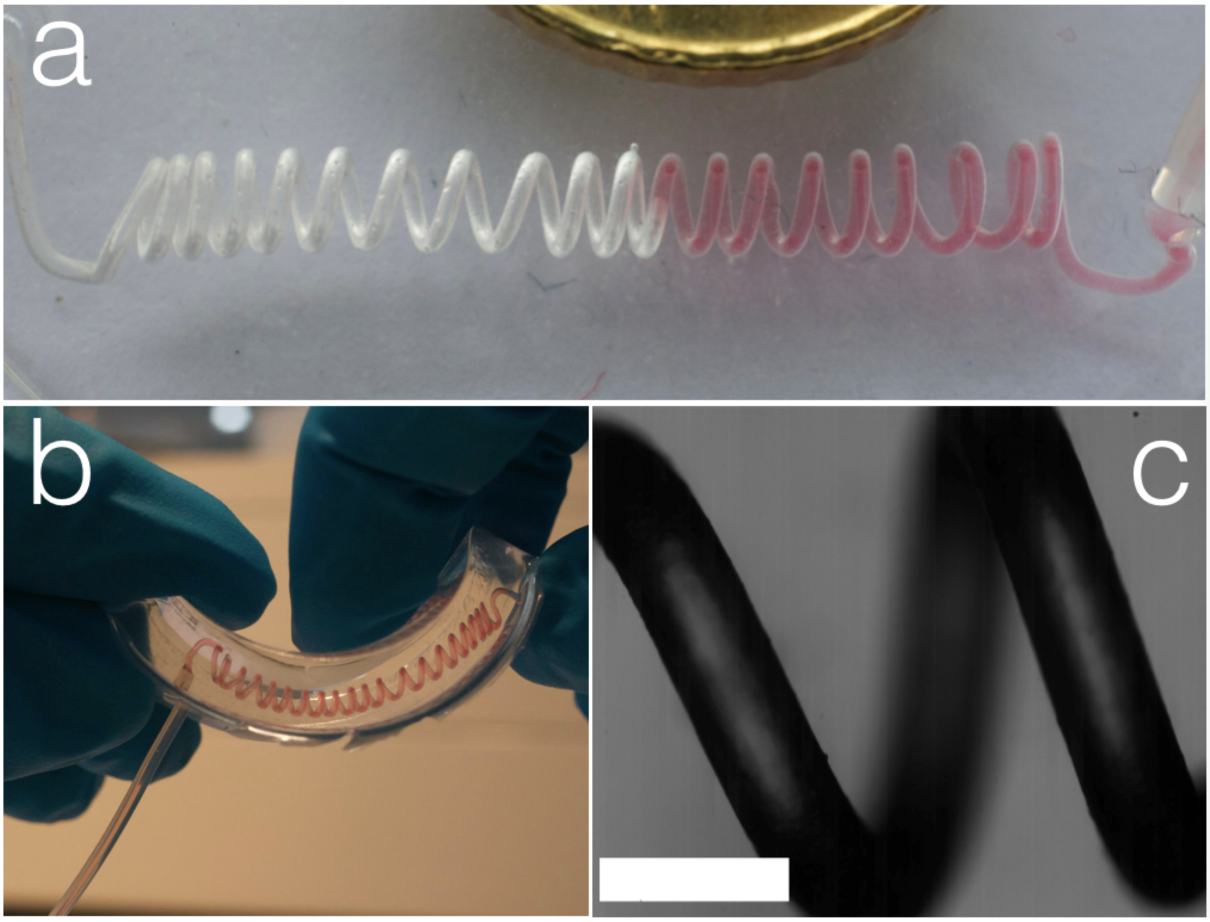

Figure S4. a) A 3D spiral microchannel and b) its elasticity inside the PDMS. c) Bright-field micrograph of the channel, scale bar = 1 mm.

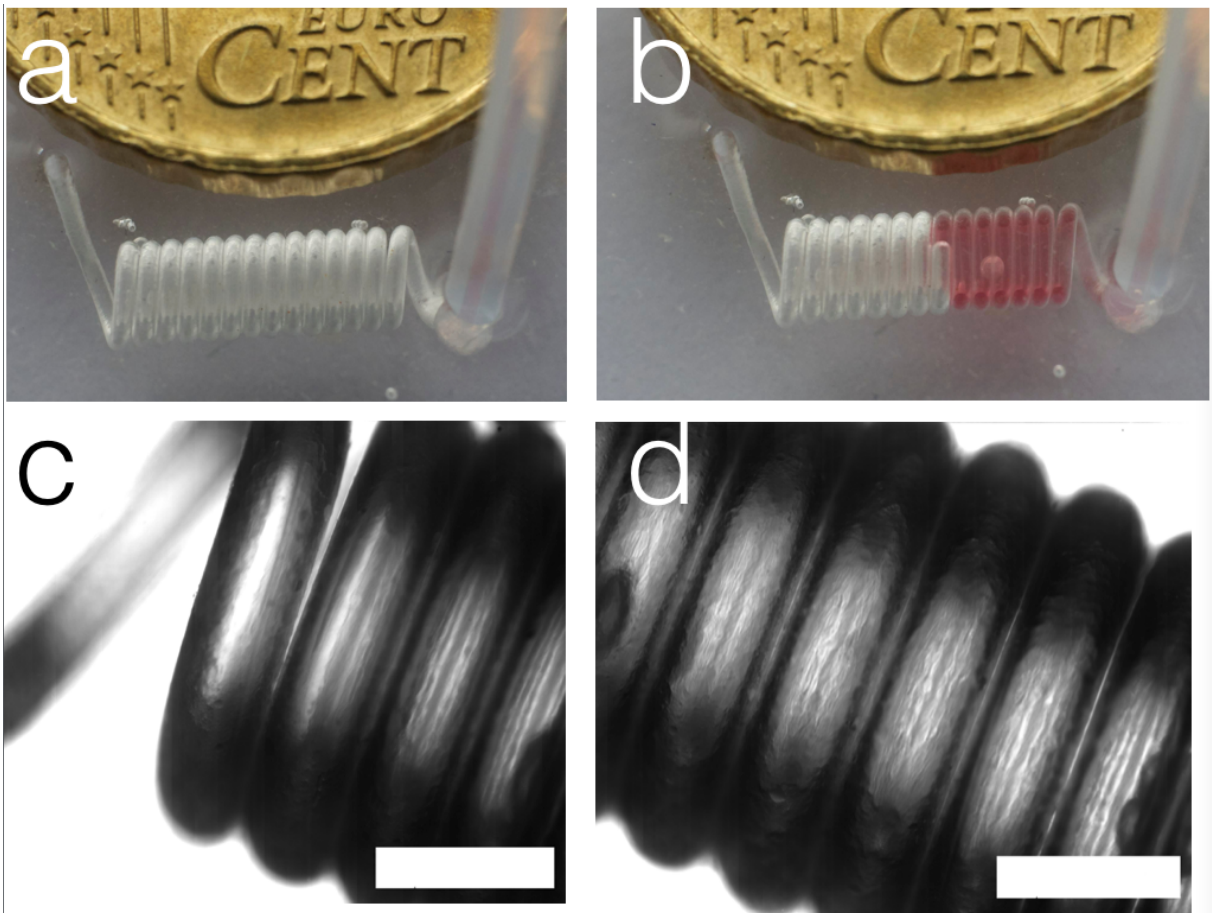

Figure S5. a,b) Tighter 3D spiral microchannel. c,d) The PDMS layer between the spiral is about 30 μm, scale bar = 1 mm.

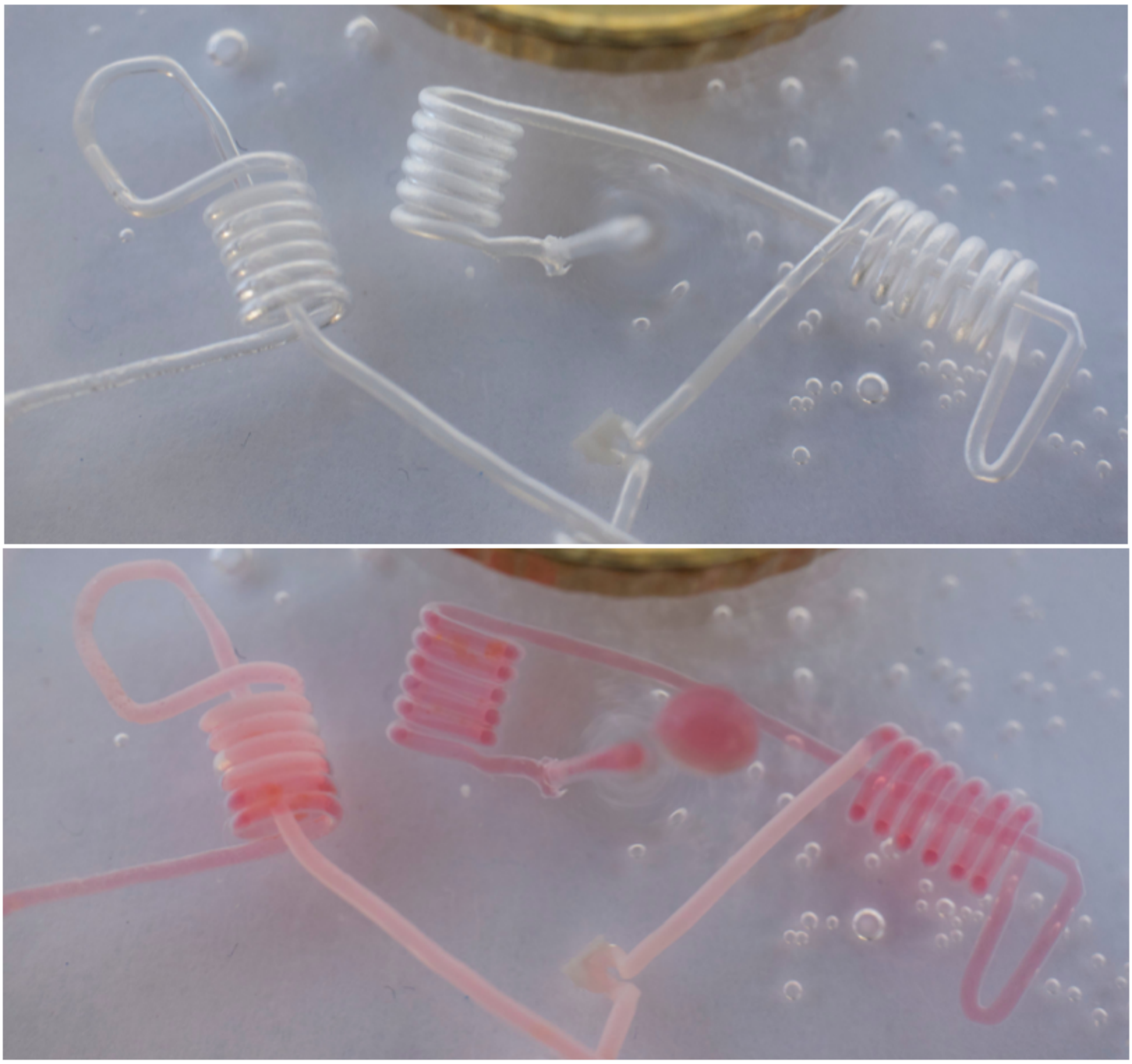

Figure S6. Complex multilevel and long 3D channels can be easily created using ABS scaffold-removal.

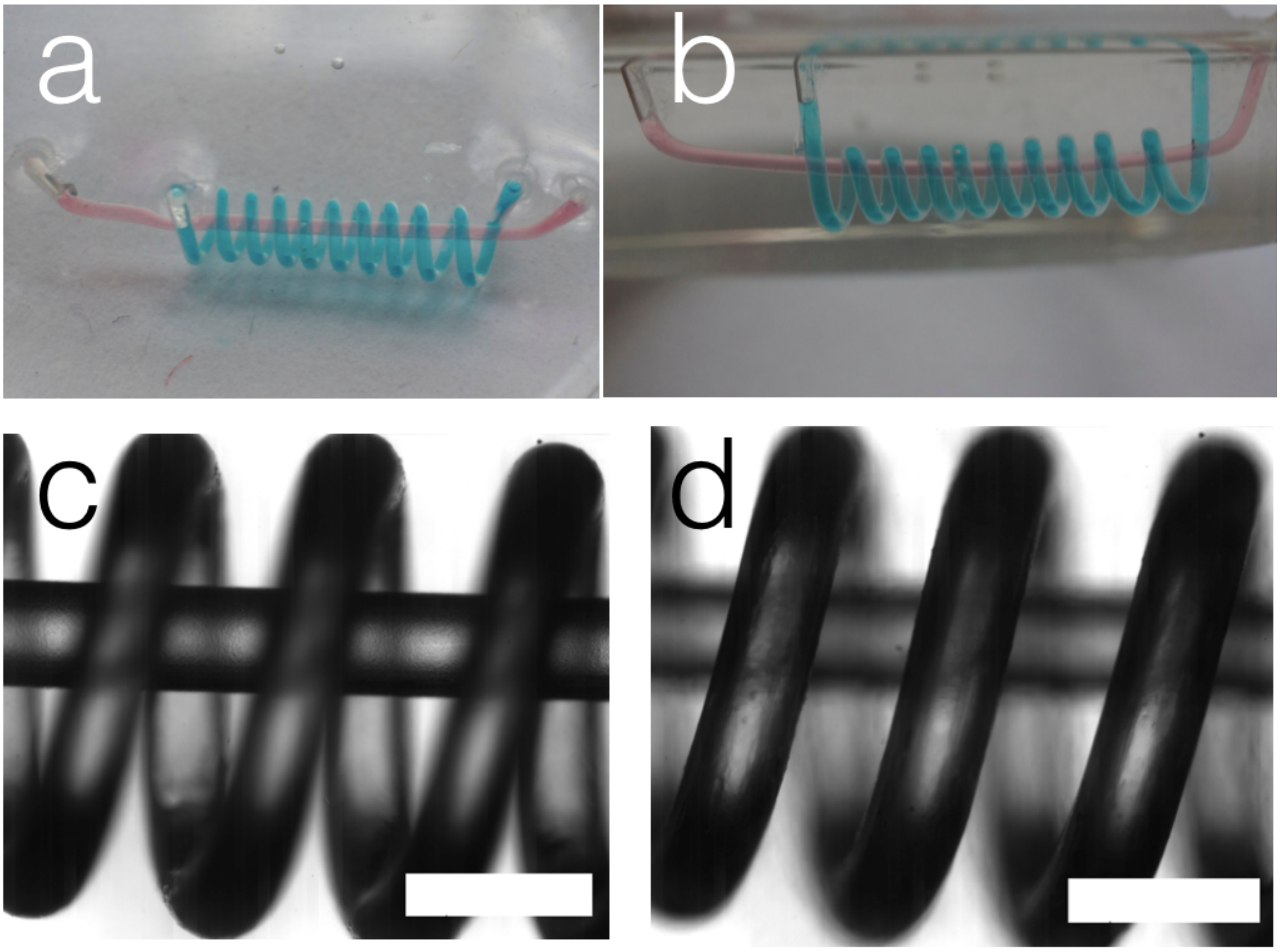

Figure S7. Using ABS scaffold-removal it is possible to create intricate structures composed of multiple microchannels. In this case (a,b) a straight channel is encompassed by another spiral channel; micrographs at different focus levels (c,d) of the two channels. Scale bar = 1 mm.

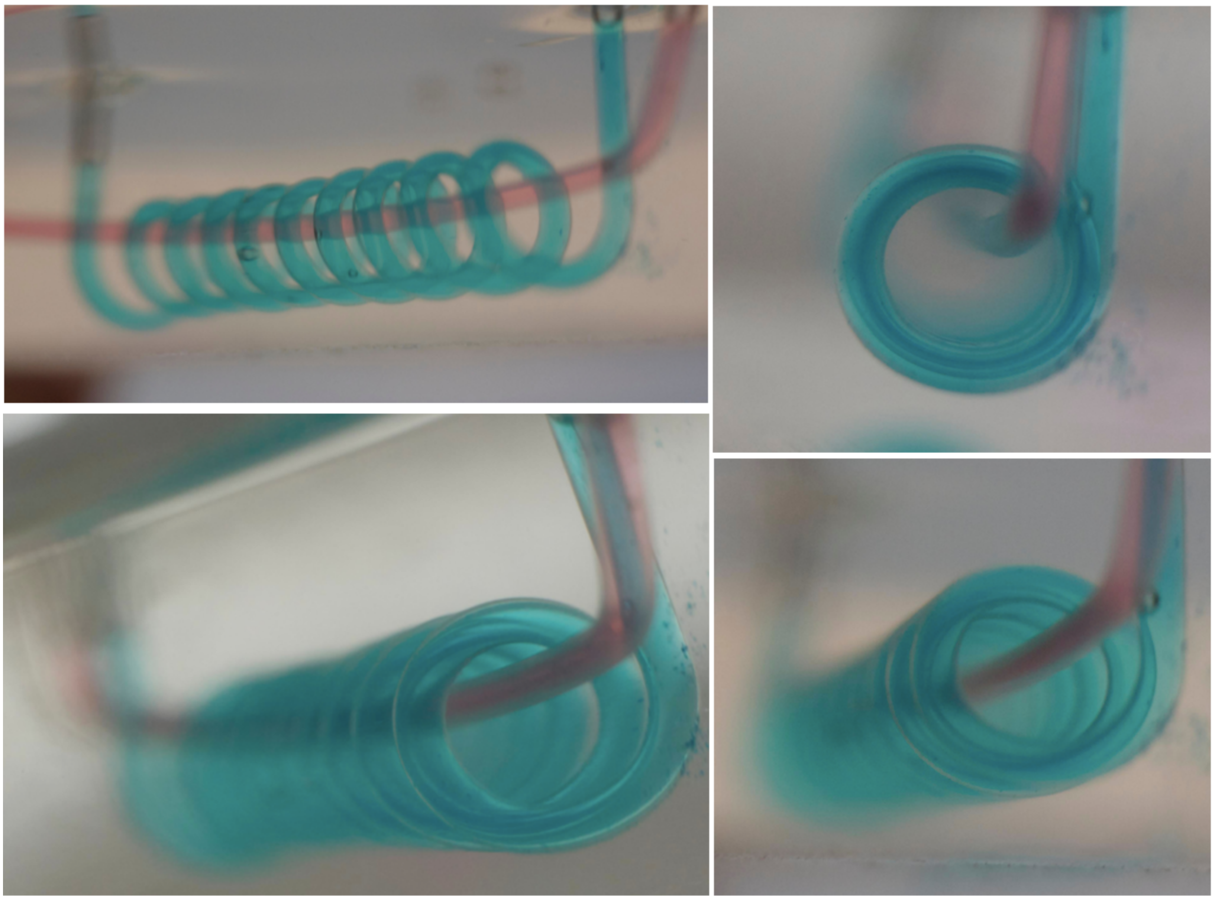

Figure S8. Close-up pictures of the microfluidic channels. Channels size = 500 μm.

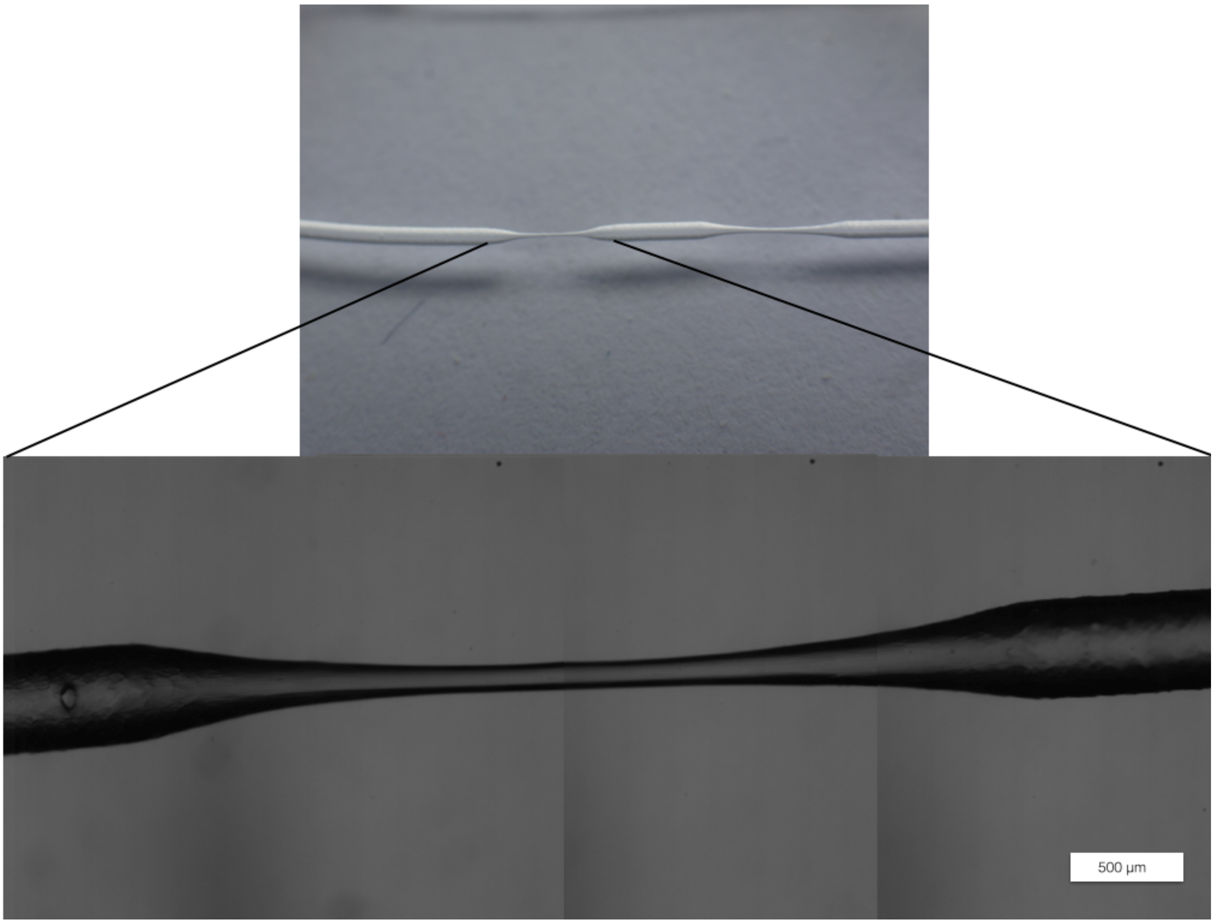

Figure S9. Single channel with different diameters (500 and 90 μm).

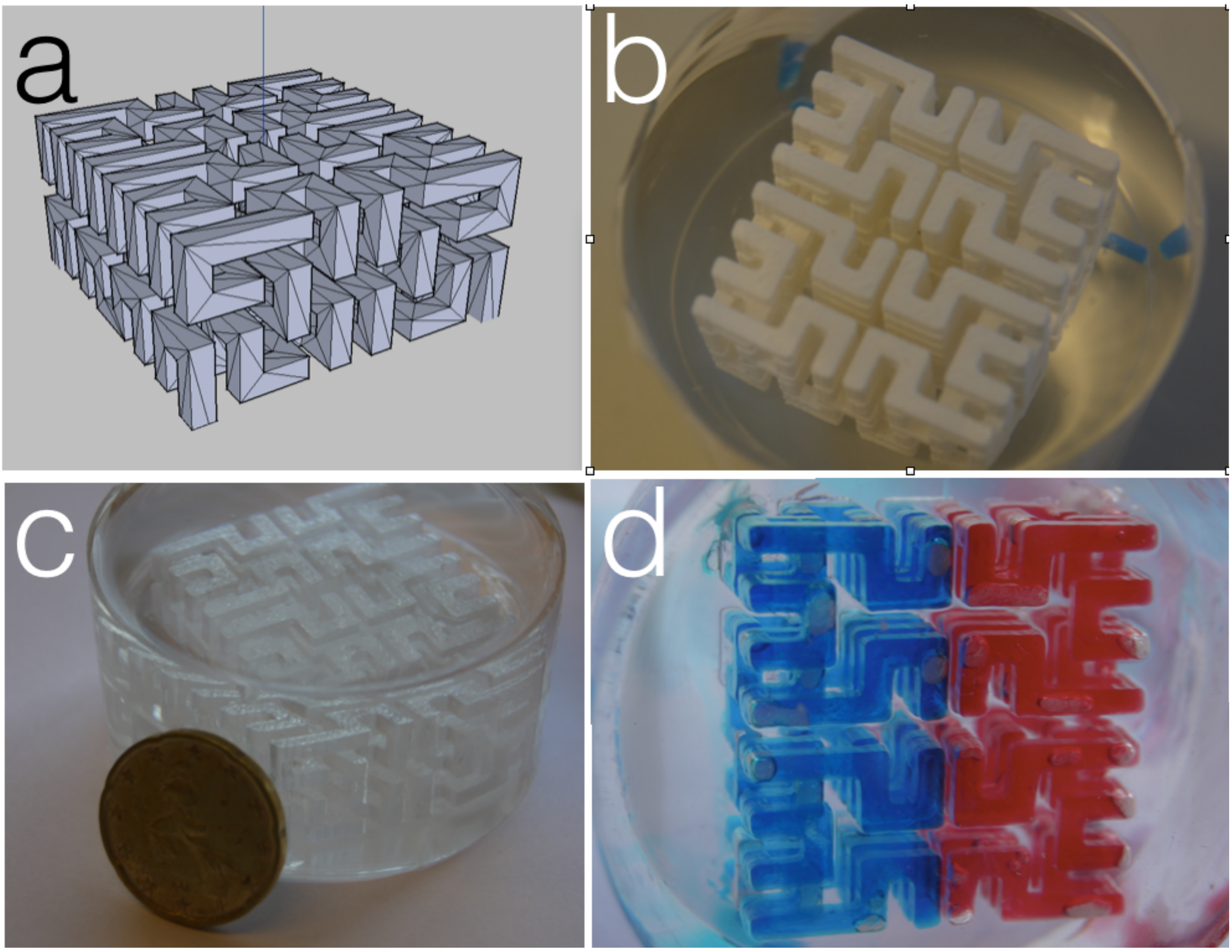

Figure S10. A 3D model (a) based on the Hilbert cube was 3D printed with ABS using fused deposition modeling, suspended in PDMS and the latter was cured (b). The ABS was then removed with dichloromethane and acetone baths, (c) forming a working microfluidic device (d). Channel diameter = 2 mm.

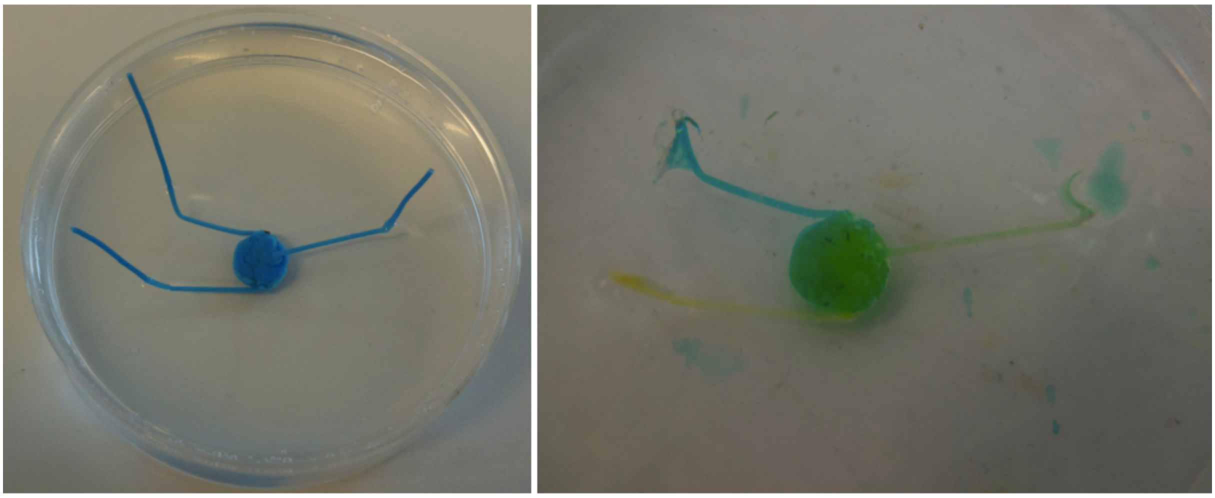

Figure S11. External components in a cavity bigger than the size of the channels guiding to it, so called “ship-in-a-bottle”, can be obtained in a simple step. For example we inserted a 1x1 mm stirring bar in an ABS disk and connected this with three ABS 500 μm filaments. After curing the PDMS and removing the ABS plastic with acetone, the stirring bar is released in the formed chamber.

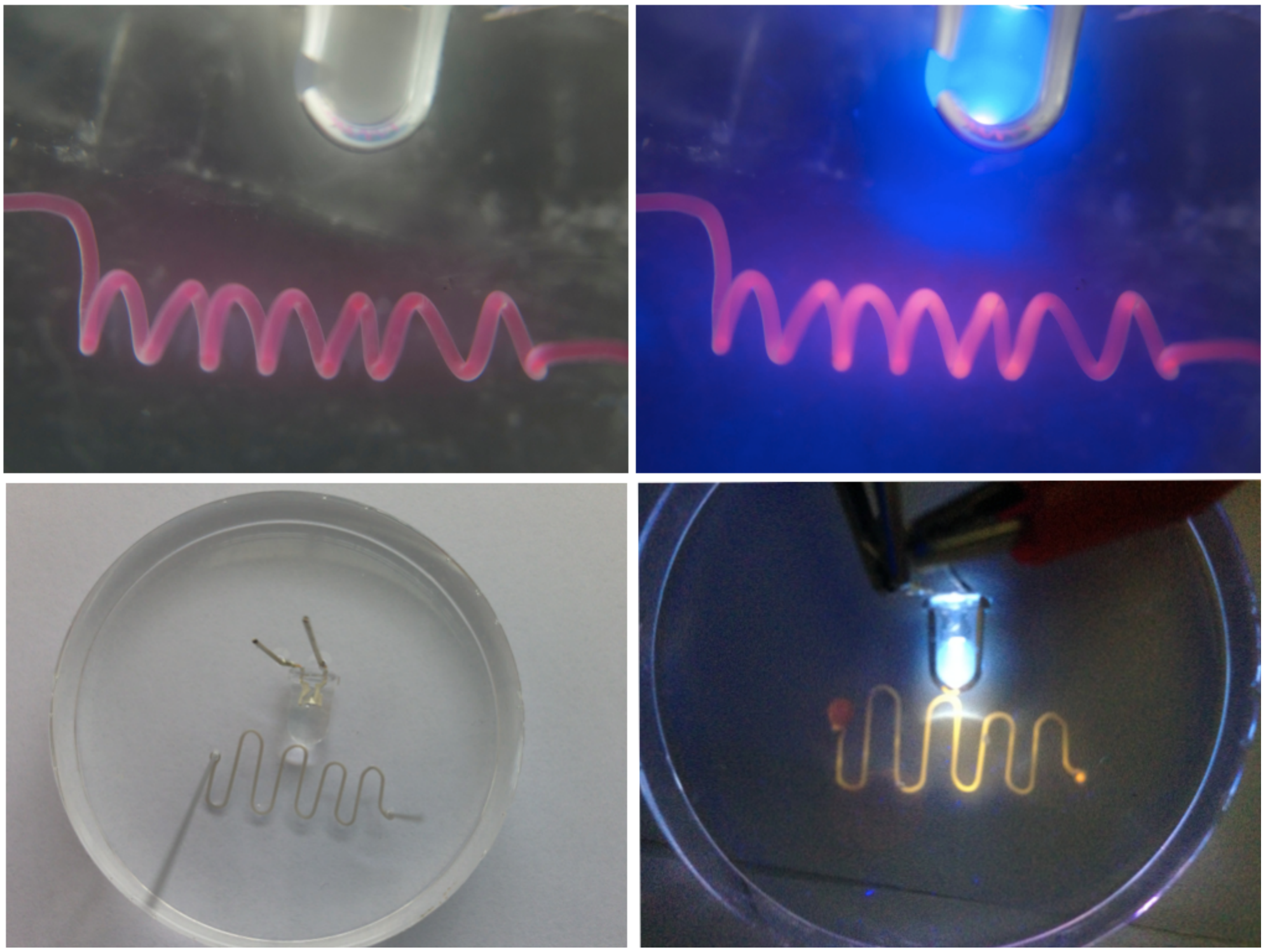

Figure S12. Electronic components can be directly inserted in the PDMS microfluidic chip during the manufacturing. The PDMS is not conductive and the subsequent immersion in acetone is not affecting the electric components inside the PDMS. Here a 390 nm LED is embedded in the chip exciting a Rhodamine B solution inside the channels.

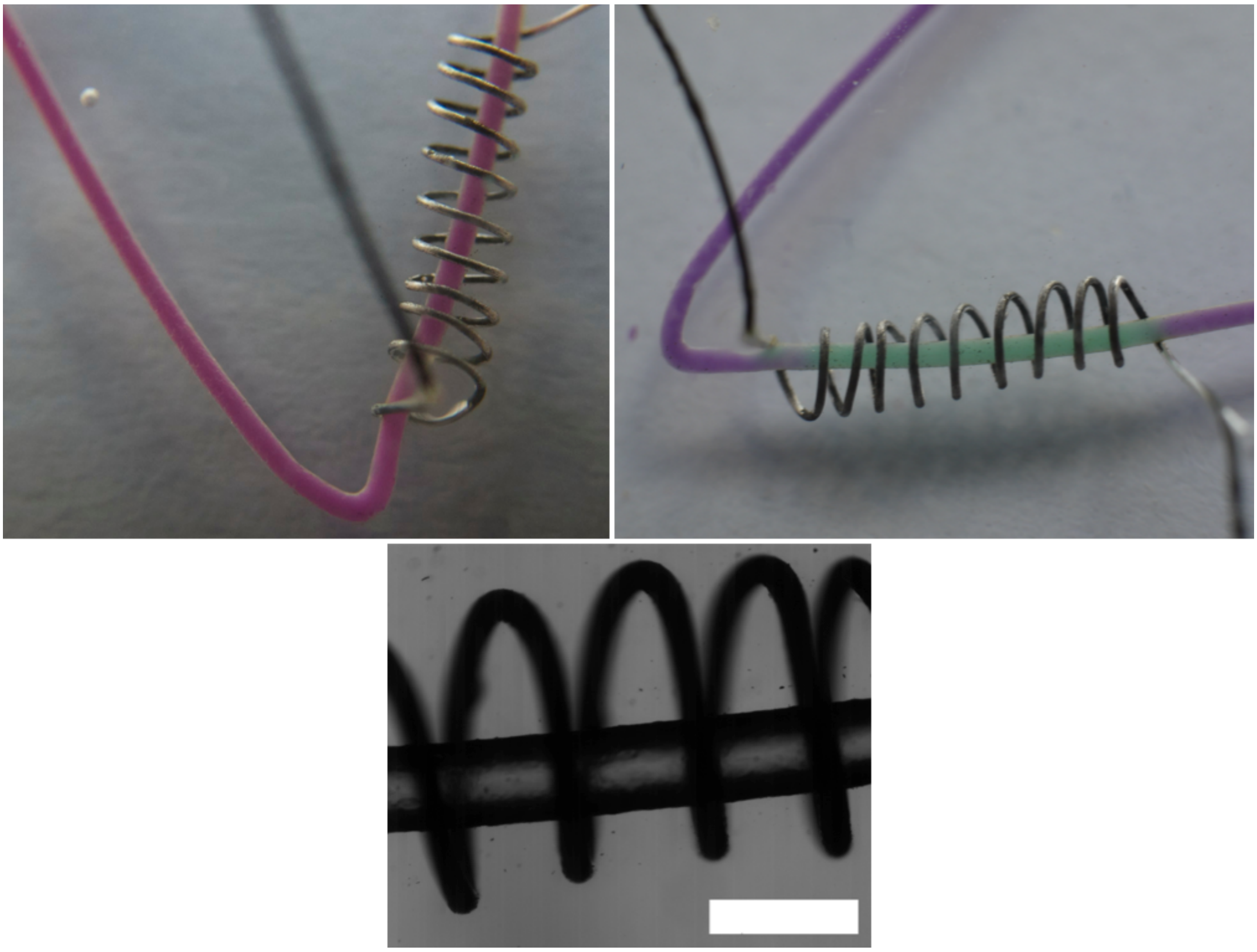

Figure S13. A 200 μm resistance wire (nichrome) was embedded in the chip for selectively heating part of the microfluidic chip. Here part of the microfluidic channel is enveloped in a spiral of resistance wire. A theromochromic dye (color changing temperature: 27 °C) was then dissolved in water as temperature indicator. When a small voltage was applied to the the wire (c.a. 1.2 V; 0.35 A) the changing color of the thermochromic dye proved the selective heating. Scale bar in the micrograph = 1 mm.

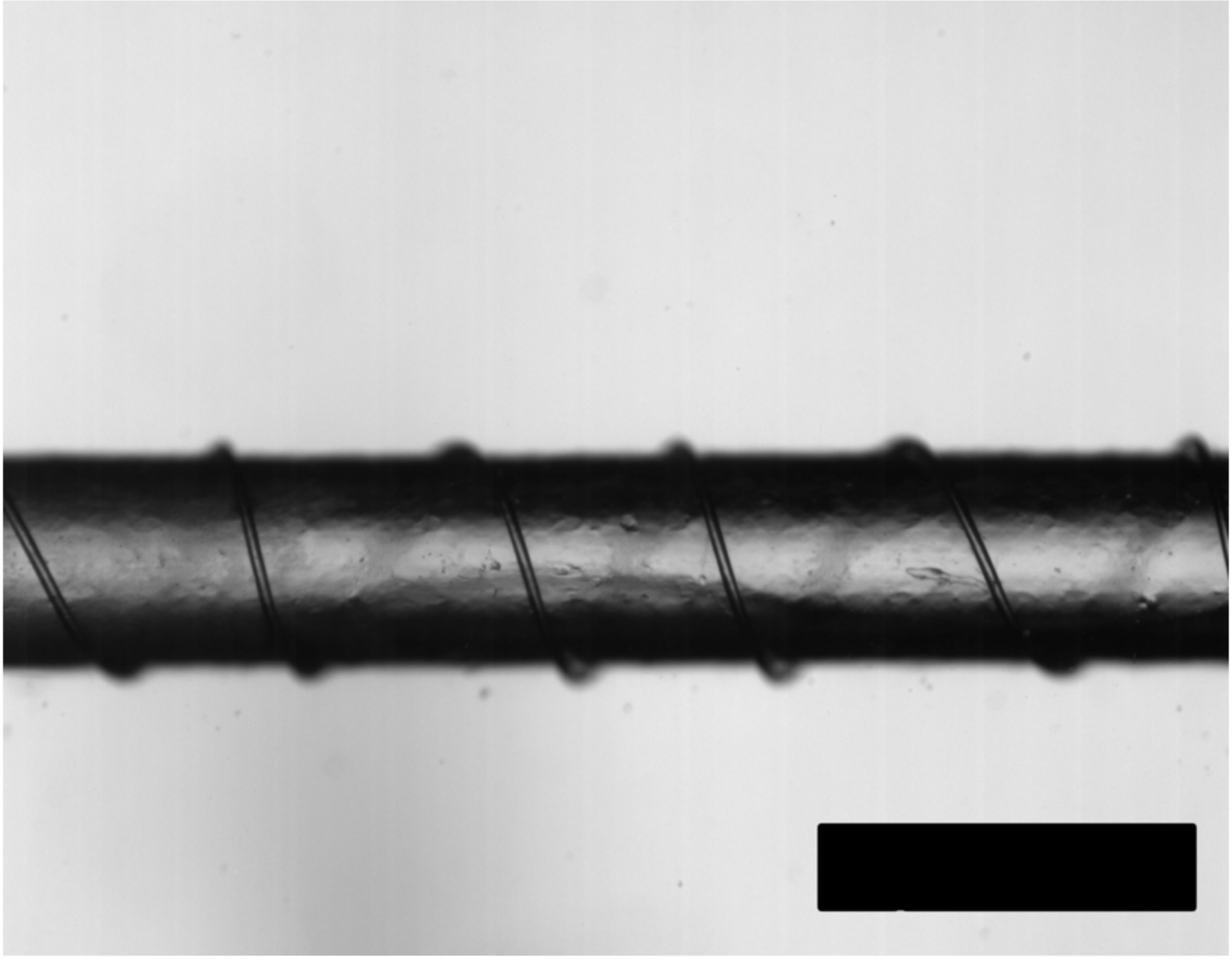

Figure S14. Small components can also be easily embedded in the design. Here a 32 μm copper wire is enveloping the microfluidic channel and used as NMR microcoil. Scale bar = 1 mm.

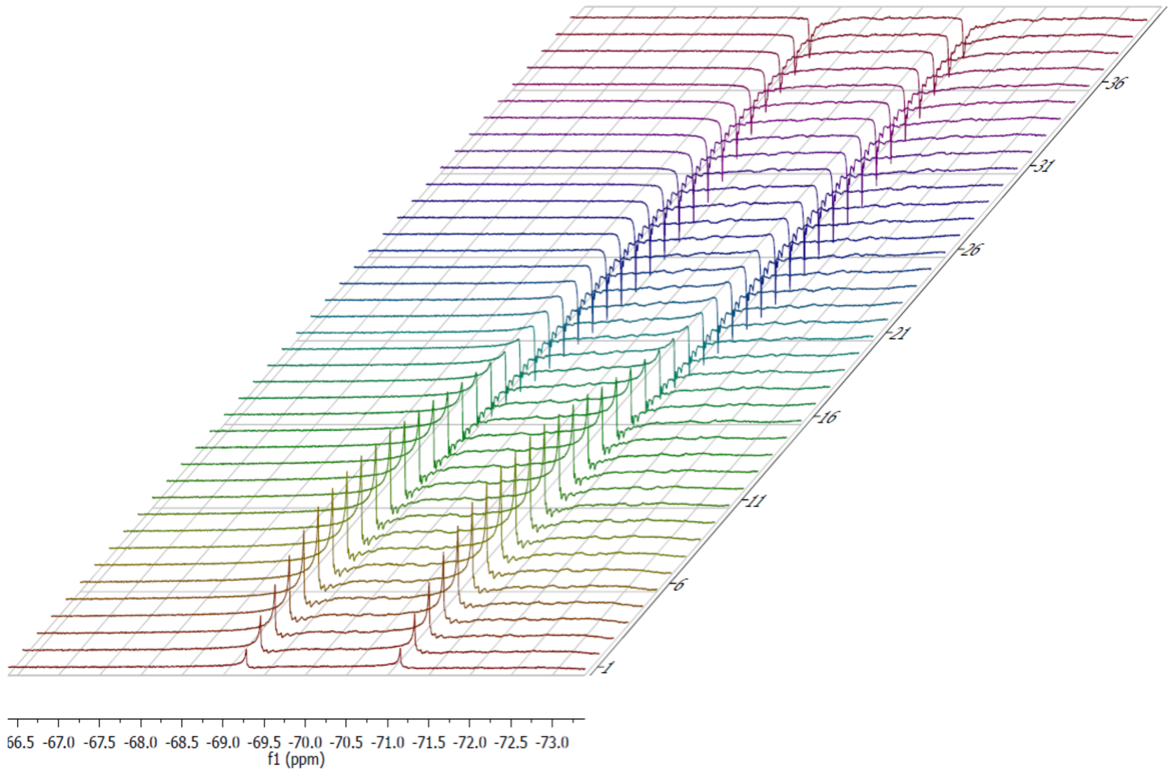

Figure S15. Nutation experiment at 376 MHz (^{19}F) on the doublet signal of the PF_6 anion of a $NaPF_6$ sample in water. The 90° pulse obtained was 10 µs with attenuation of the transmitter amplifiers of 12 dB.

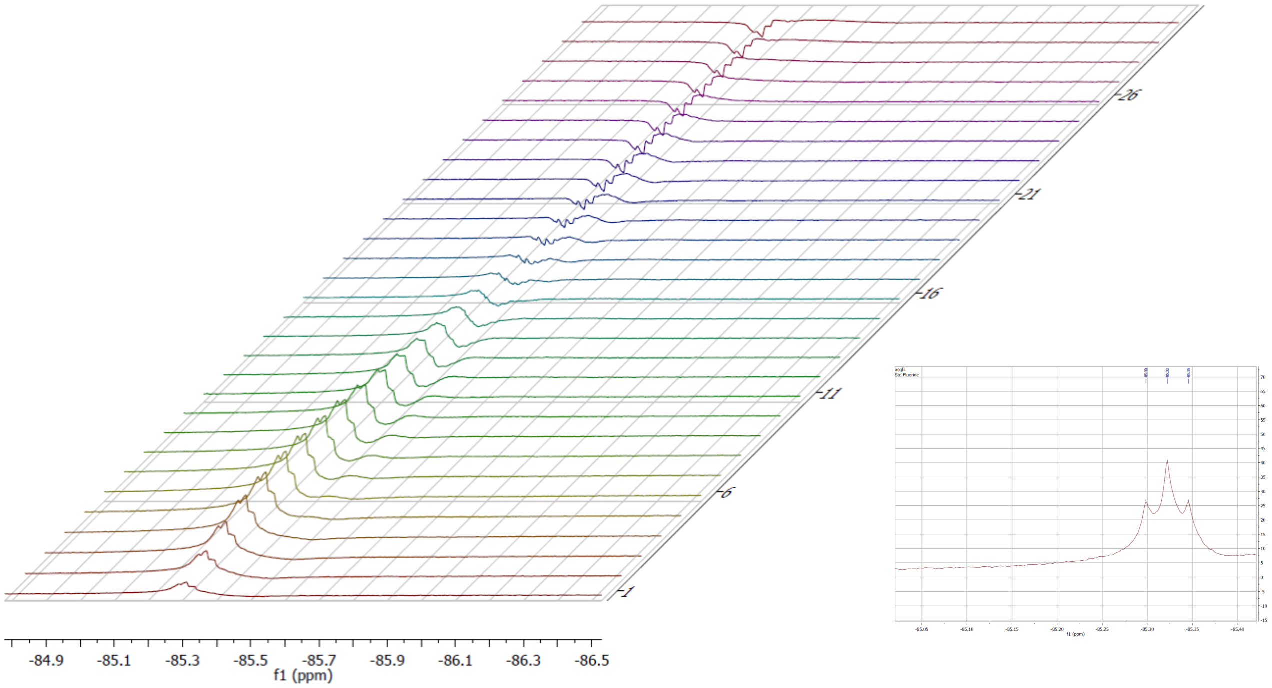

Figure S16. Nutation experiment for the trifluoethanol sample (left) and enlarged peak showing the 8 Hz $^{3}J_{HF}$ spin-spin coupling between the fluorine and proton nuclides.

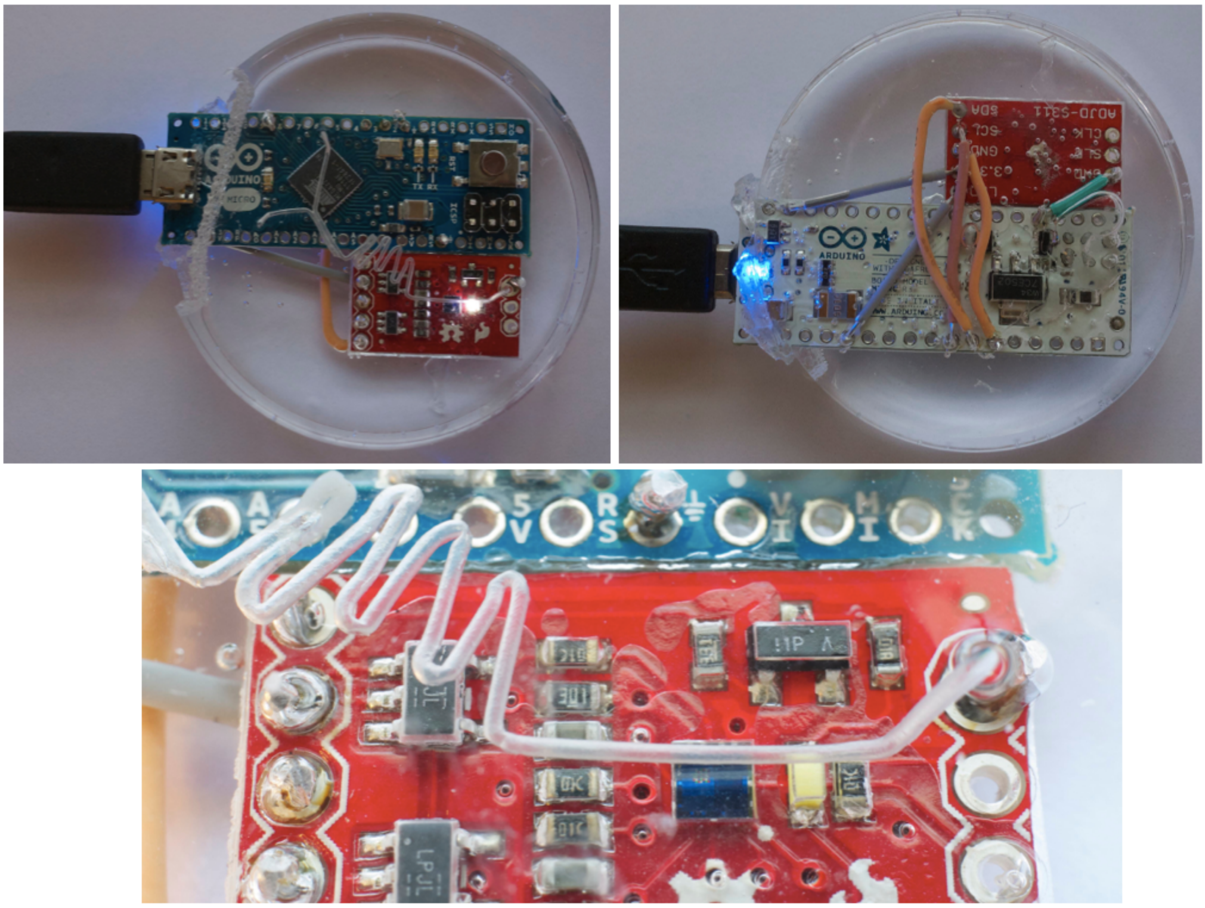

Figure S17. Even complex electronics can be embedded in PDMS chips. Here a RGB sensor (ADJDS-311) attached to an arduino micro are embedded in a microfluidic chip. The RGB sensor was connected to the Arduino board and the contacts were soldered with a iron solder. Then our procedure for creating the channel was performed. Within the acetone step, the microfluidic channel was created on top of the sensor. This particular sensor was too big for the size of the channel, therefore a 2x2mm chamber was created afterwards on top of the sensor. Three colored dyes were used to test the functionality of the electronics (see Figure 18).

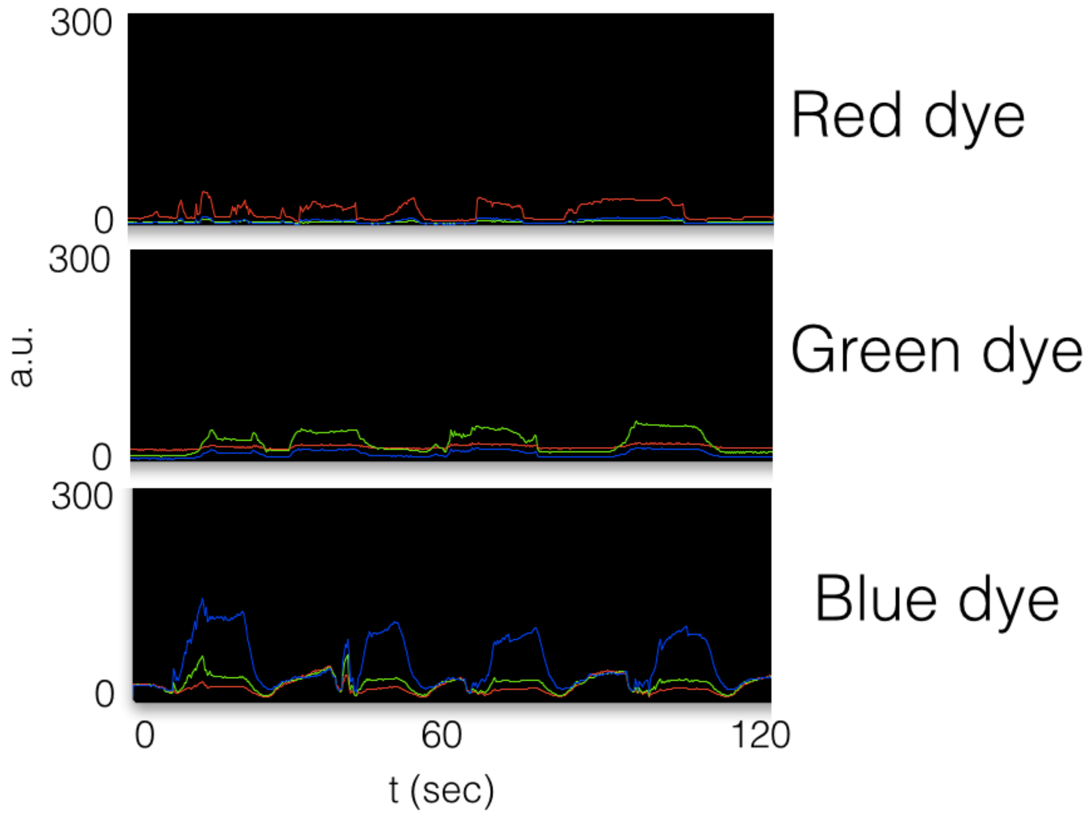

Figure S18. Three dyes were flowed separately and intermittently in the microfluidic chip, an halogen light was used for illuminating the flow cell. The RGB sensor detected the different dyes proving that the sensor and the microcontroller can be easily embedded in the PDMS microfluidic chip using the ABS scaffold-removal method.
The Arduino micro was programmed with the Arduino IDE 1.0.5.
For graph the results in real time Processing (ver. 2.2.1) was used.
